# Novel Vaccines Targeting the Highly Conserved SARS-CoV-2 ORF3a Ectodomain Elicit Immunogenicity in Mouse Models

**DOI:** 10.3390/vaccines13030220

**Published:** 2025-02-22

**Authors:** Jacob Meza, Elizabeth Glass, Avinaash K. Sandhu, Yangchen Li, Styliani Karanika, Kaitlyn Fessler, Yinan Hui, Courtney Schill, Tianyin Wang, Jiaqi Zhang, Rowan E. Bates, Alannah D. Taylor, Aakanksha R. Kapoor, Samuel K. Ayeh, Petros C. Karakousis, Richard B. Markham, James T. Gordy

**Affiliations:** 1 Department of Molecular Microbiology and Immunology, Johns Hopkins Bloomberg School of Public Health, Baltimore, MD 21205, USA; jmeza2@jhu.edu (J.M.); axs1234@case.edu (A.K.S.); yli546@alumni.jh.edu (Y.L.); kcomstock29@aol.com (K.F.); huiyinan@grinnell.edu (Y.H.); cschill6@jhmi.edu (C.S.); twang148@jhmi.edu (T.W.); jzhan346@alumni.jh.edu (J.Z.); rbates15@jh.edu (R.E.B.); atayl149@jh.edu (A.D.T.); petros@jhmi.edu (P.C.K.); 2 Center for Tuberculosis Research, Division of Infectious Diseases, Department of Medicine, Johns Hopkins University School of Medicine, Baltimore, MD 21287, USA; skarani1@jhmi.edu (S.K.); aak4006@med.cornell.edu (A.R.K.); sayeh1@alumni.jh.edu (S.K.A.)

**Keywords:** ORF3a, SARS-CoV-2, COVID-19, MIP3α/CCL20, DNA vaccine, KLH, peptide vaccine, evolutionarily conserved antigen, T-cell response, antibody response

## Abstract

Background: The majority of antigen-based SARS-CoV-2 (SCV2) vaccines utilized in the clinic have had the Spike protein or domains thereof as the immunogen. While the Spike protein is highly immunogenic, it is also subject to genetic drift over time, which has led to a series of variants of concern that continue to evolve, requiring yearly updates to the vaccine formulations. In this study, we investigate the potential of the N-terminal ectodomain of the ORF3a protein encoded by the *orf3a* gene of SCV2 to be an evolution-resistant vaccine antigen. This domain is highly conserved over time, and, unlike many other SCV2 conserved proteins, it is present on the exterior of the virion, making it accessible to antibodies. ORF3a is also important for eliciting robust anti-SARS-CoV-2 T-cell responses. Methods: We designed a DNA vaccine by fusing the N-terminal ectodomain of *orf3a* to *macrophage-inflammatory protein 3α* (MIP3α), which is a chemokine utilized in our laboratory that enhances vaccine immunogenicity by targeting an antigen to its receptor CCR6 present on immature dendritic cells. The DNA vaccine was tested in mouse immunogenicity studies, vaccinating by intramuscular (IM) electroporation and by intranasal (IN) with CpG adjuvant administrations. We also tested a peptide vaccine fusing amino acids 15–28 of the ectodomain to immunogenic carrier protein KLH, adjuvanted with Addavax. Results: The DNA IM route was able to induce 3a-specific splenic T-cell responses, showing proof of principle that the region can be immunogenic. The DNA IN route further showed that we could induce ORF3a-specific T-cell responses in the lung, which are critical for potential disease mitigation. The peptide vaccine elicited a robust anti-ORF3a antibody response systemically, as well as in the mucosa of the lungs and sinus cavity. Conclusions: These studies collectively show that this evolutionarily stable region can be targeted by vaccination strategies, and future work will test if these vaccines, alone or in combination, can result in reduced disease burden in animal challenge models.

## 1. Introduction

The vaccine efforts during the height of the pandemic were instrumental in decreasing the morbidity and mortality of the SARS-CoV-2 outbreak and gave rise to several vaccines to combat the disease—BNT162b2 (Pfizer-BioNTech), mRNA-1273 (Moderna), Ad26.COV2.S (Janssen), and NVX-CoV2373 (Novavax) [1,2,3]. All of the currently FDA-approved vaccines utilize the Spike (S) glycoprotein of the SARS-CoV-2 virus as an antigen [1,3]. However, as a result of the emergence of mutation-carrying variants of concern (VOC), it has become imperative for researchers to identify an alternative antigen from a more conserved portion of the virus that is still accessible to antibodies.

The SARS-CoV-2 viral genome is approximately 29.7 kb and encodes a total of 29 proteins that include 16 nonstructural proteins, 4 structural proteins, and 9 accessory open reading frames (ORFs) [4]. Among them is the protein ORF3a, which has been discovered to be the largest accessory protein of the SARS-CoV-2 virus. The ORF3a protein has been assigned significant roles in the viral pathogenesis and disease severity of COVID-19 [4,5], mainly in the form of cell membrane permeability, Ca^2+^ homeostasis, viral entry and replication, and release of viral particles [5]. This impact on COVID-19 disease severity comes in the form of a cytokine storm induction, where ORF3a has been observed to induce a pro-inflammatory immune response in infected cells, activating the NOD-like receptor protein 3 (NLRP3) inflammasome and creating a hyper-inflammatory response [5].

The rationale for targeting the ORF3a protein follows the recent literature regarding its significant contribution to disease severity and viral pathogenesis. Most notably, the 1-34 N-terminal ectodomain of ORF3a has been found to be highly conserved and is a viable therapeutic target due to its presence on the surface of the virion. Historically, both cell- and humoral-based immunity have been essential for the control of infection [6,7,8], and since this region is exposed on the virion surface, it is accessible to antibodies, unlike other internal nonstructural proteins that are highly conserved, such as the nucleoprotein (NP). Further, ORF3a was found to contain immunodominant T-cell responses in people [9,10,11], and vaccines targeting residues 15–28 in the SARS virus were able to induce antibody responses [12]. A primary hypothesis of this work is that the ectodomain of ORF3a, due to its availability on the surface and uniformity across strains, presents a vaccine target that is superior to other conserved proteins, like NP, that are not present on the virion surface and that is a viable alternative to Spike, which requires yearly changes to account for genetic drift across strains.

CCL20, also known as macrophage inflammatory protein-3 alpha (MIP-3α), is the only chemokine that has high specificity for the chemokine receptor CCR6, which is highly expressed on several human cell types including immature dendritic cells (iDCs), effector/memory CD8+ and CD4+ T cells, and interleukin-17 (IL-17)-producing T cells (TH_17_) [13]. The ligand–receptor pair of CCL20-CCR6 is also responsible for the chemoattraction of iDCs and plays a role in the pathogenesis and pathology of several inflammatory diseases, including cancer, psoriasis, and rheumatoid arthritis [13,14]. Therapeutically, MIP3α has also been utilized to enhance the immunogenicity of DNA vaccination when fused to an antigen by increasing DC trafficking, targeting, and activation [15,16,17]. Previous disease models using *Haemophilus influenzae*, green fluorescent protein reporter systems, melanoma, *Mycobacterium tuberculosis*, malaria (*Plasmodium* spp.), and the SARS-CoV-2 receptor-binding domain have all found MIP3α to be an effective iDC-targeting agent that has the potential to be utilized to achieve antigen-specific protective immunity [18,19,20,21,22,23,24,25].

Here, we present our immunological findings of vaccines in murine models targeting the ORF3a ectodomain, including a DNA vaccine expressing *MIP3α-ORF3a_6-34_*, administered intramuscularly (IM) with electroporation or intranasally with CpG adjuvant, as well as a peptide vaccine of ORF3a_15-28_ fused to keyhole limpet hemocyanin (KLH), administered systemically with Addavax adjuvant. Our results indicate that the DNA formulation elicits ORF3a-specific T-cell responses in the spleen with IM and in the lung with IN immunizations. Our studies further indicate that the peptide vaccine elicits an anti-ORF3a antibody response in the serum as well as in the lung and sinuses. We show that this highly conserved, antibody-accessible region of SARS-CoV-2 ORF3a can be targeted by vaccines eliciting T-cell and antibody-based responses in the lung.

## 2. Materials and Methods

### 2.1. Animals

C57BL/6 or BALB/C mice (4–5 weeks old) were purchased from Charles River Laboratories (Wilmington, MA, USA) and maintained in a pathogen-free micro-isolation facility in accordance with the National Institutes of Health guidelines for the humane use of laboratory animals. Strain and Sex are noted in the figure legends. All experimental procedures involving mice were approved by the IACUC of Johns Hopkins University (Protocol Number: MO23H131). The experiments began after the mice reached 6 to 10 weeks of maturity.

### 2.2. DNA Vaccine Plasmid Construction and Verification

A pUC57 plasmid containing DNA encoding mouse codon-optimized *MIP3α-ORF3a_6-34_* was purchased from GenScript (Piscataway, NJ, USA) and was reconstituted (200 ng/µL) and transformed into DH5-α E. coli cells (Invitrogen™ Thermo Fisher Scientific (TFS), Waltham, MA, USA). The transformed bacteria were selected with ampicillin (100 mg/mL) (Millipore Sigma, Burlington, MA, USA). DNA was then extracted from the transformed bacteria using a Qiagen (Germantown, MD, USA) QIAprep Spin Miniprep Kit. DNA quality, amount, and correctness were verified by agarose gel electrophoresis, restriction enzyme analysis, and NanoDrop (TFS) spectrophotometry. The *MIP3α-ORF3a_6-34_*-containing plasmid was then cut using HindIII and BamHI (New England BioLabs, Inc. (NEB), Ipswich, MA, USA) restriction enzymes and ligated into a previously generated pSecTag2b plasmid [22]. The vaccine region begins at amino acid 6 due to methionines at residues 1 and 5 that could have acted as alternative start sites.

*c-Myc* tag and poly-histidine tag regions (Myc-His) were synthesized to help detect the expressed protein using gBlocks Gene Fragments from Integrated DNA Technologies (Coralville, IA, USA). The sequence was as follows: 5′ GGTGAACGAGCGGCGATACGCGGATCCAGATCCGCAGAAGAACAGAAACTGATCTCAGAAGAGGATCTGGCCCACCACCATCACCATCACTAAGAATTCACTCGGATCTTACACTCTAGCCGGACATGC 3′. The Myc-His tag fragments were then cut using BamHI and EcoRI restriction enzymes (NEB) and cloned into the previously ligated pSecTag2b product. A second version of the vaccine was created in the same way but with an additional PADRE sequence (AKFVAAWTLKAAA) between the antigen and the tags. Pan-HLA-DR Epitope (PADRE) is a 13-amino-acid peptide that enhances immune responses by recruiting and activating CD4+ T cells, thereby boosting antigen-specific CD8+ T cell activity and increasing B cell-mediated IgG production across multiple disease models [26,27,28,29,30,31]. In line with our laboratory’s efforts to achieve both higher levels of T cell immunity and provide protection via an antibody response, we conjugated an additional peptide to our MIP-3αORF3a_6-34_ fusion vaccine to elicit a stronger immune response.

The resulting ligation was then transformed into DH5-α E. coli cells (Invitrogen, Waltham, MA, USA). The bacteria were then selected with ampicillin (100 mg/mL). DNA was extracted from the bacteria containing the ligated product using Qiagen EndoFree Plasmid Kits and was diluted with endotoxin-free 1X PBS to 400 ng/µL. DNA quality, amount, and correctness were verified once again by agarose gel electrophoresis, restriction enzyme analysis, and NanoDrop spectrophotometry.

### 2.3. Mammalian Cell Transfection

HEK293T cells (American Type Culture Collection, Manassas, VA, USA) were cultured at 37 °C and 5% CO_2_ using DMEM (Gibco™, TFS, Grand Island, NY, USA) media, containing 4.5 g/L glucose, 4% L-glutamine, and 110 mg/L sodium pyruvate, with additions of 10% FBS (Heat inactivated, Millipore Sigma) and 1% penicillin and streptomycin (10,000 U/mL, Gibco™, TFS). HEK293T cells were plated at a seeding density of 2 × 10^5^ cells/well into a Falcon Multiwell 12-well tissue culture treated plate (Corning, Inc.; Corning, NY, USA). The plate was kept at 37 °C and 5% CO_2_ until the cell culture reached approximately 70% confluency. The cells were then transfected with 5 µg/well of the *MIP3α-ORF3a_6-34_* DNA vaccine using the Lipofectamine 3000 Reagent Protocol supplied by Invitrogen and then returned to the incubator at 37 °C and 5% CO_2_ for 72 h.

### 2.4. Western Blot

After 72 h, the proteins from cell lysates and culture supernatants were collected. The cell supernatants were then concentrated using an Amicon-Ultra 4kd centrifuge tube (Millipore Sigma) and spun for an hour. Both cell lysates and culture supernatants were then run on precast TGX Gels (Bio-Rad, Hercules, CA, USA) at 150 V for 30 min and transferred to a nitrocellulose membrane (Bio-Rad). The membrane was then blocked with a 5% milk solution containing 1X TBS (Quality Biological, Gaithersburg, MD, USA), probed with anti-C-myc (Cell Signaling Technology, Danvers, MA, USA) at RT for 2 h at 1:5000 dilution, washed, probed with AP-conjugated goat anti-mouse antibody (Jackson ImmunoResearch Laboratories, Inc., West Grove, PA, USA) at 1:1000 dilution for 1 h, washed, and visualized with NBT-BCIP reagent (Sigma Aldrich, St. Louis, MO, USA). The blot image was edited to remove lanes of a different project. The unedited image is in Supplemental Figure S1. The blots here are qualitative in nature and, therefore, do not have density estimates.

### 2.5. Vaccine Administration

*MIP3α-ORF3a* vaccine formulations were diluted in 1X endotoxin-free PBS to 400 ng/µL and were administered intramuscularly (IM) by injection of 50 µL (20 µg) into the right gastrocnemius muscle of each mouse. The saline group received 50 µL of 1X endotoxin-free PBS. The vaccinations were given to all three groups at 2-week intervals for a total of three doses and were accompanied by electroporation [32,33]. Blood was obtained by tail vein nicking just prior to each vaccination and processed for ELISA-based detection of anti-ORF3a antibodies. Local electroporation was administered using an ECM830 square wave electroporation (EP) system (BTX Harvard Apparatus Company, Holliston, MA, USA). Each of the two-needle array electrodes delivered 15 pulses of 72 V (a 20 ms pulse duration at 200 ms intervals). The weights of each mouse were also recorded prior to each vaccination (Supplemental Figure S2). For the purposes of this study, both vaccine groups were combined since the PADRE formulation showed no immunological difference from the original *MIP3α-ORF3a* vaccine, likely due to an unanticipated shielding or interaction with other vaccine regions, potentially due to the likely dimerization we observed in the Western blot (Supplemental Figure S3). Pilot experiments with the same protocol, except for adding 50 μg of CpG-B (ODN 1826, Adipogen, San Diego, CA, USA), CpG-C (ODN 2395, Adipogen) or a STING agonist (2′3′-c-di-AM(PS)2 (Rp,Rp), Invivogen, San Diego, CA, USA) at the time of vaccination, were also performed.

For the mice immunized intranasally, the mice were first anesthetized by inhaled vaporized isoflurane and then were given approximately 100 μL total volume (50 per nostril) dropwise by pipette, as previously described [22,24]. The vaccines were prepared and mixed evenly by inversion with CpG-B or 1X PBS. Final concentrations of 2 mg/mL vaccine (200 μg total) and 200 μg/mL CpG (20 μg total) were administered.

For the studies of mice immunized with ORF3a-KLH, the protein vaccine was created and verified by GenScript, with the alpha strain ORF3a 15–28 region (LKQGEIKDATPSDF) fused to Keyhole limpet hemocyanin (KLH) on the N-terminal side. The 15–28 region was chosen based on work with SARS showing that the region conjugated to a carrier was able to induce antibody responses [12]. Quality control measures included HPLC and Mass Spectrometry analyses performed by GenScript. The vaccines were combined with an equal volume of either Addavax adjuvant (Invivogen) or 1X PBS and mixed gently. The vaccines were administered intraperitoneally at 200 μL total volume at 50 or 200 μg doses, as described in the figure.

### 2.6. Enzyme-Linked Immunosorbent Assay (ELISA)

Serum and bronchoalveolar lavage (BAL) fluid were collected as described previously [24]. Briefly, after euthanasia by high-dose Tri-bromoethanol (TFS) administration, blood was collected by cardiac puncture and was allowed to coagulate for 1 h at room temperature; the clotted blood was pelleted, and the serum supernatant was collected. Wash fluid for BAL and nasal wash was prepared as 1X PBS with 100 μM EDTA (0.5 M Corning, Glendale, AZ, USA) and 1X protease inhibitors (200X PMSF, Cell Signaling Technology, Danvers, MA, USA). Post-cardiac puncture, a mouse endotracheal tube (20 G× 1 in., Kent Scientific Corp., Torrington, CT, USA) was inserted into the trachea, and 0.5 mL of wash fluid was syringe-injected into the lungs and aspirated back into the syringe. This process was repeated once if the initial yield was less than approximately 250 μL. For the nasal washes, after cardiac puncture and BAL extraction, the mouse was laid on its side, and 50 μL of the wash fluid was injected into the upper nostril by pipette and then aspirated back into the pipette from the lower nostril. The procedure was repeated once if less than 25 μL was recovered. The BAL and nasal fluids were transferred to tubes, the cells were pelleted, and the fluid supernatants were utilized.

ELISAs were performed as previously described [24]. ELISA plate wells were coated with 1 μg of ORF3a_1-34_ peptide (GenScript) overnight at 4 °C, washed with PBS-T (1X PBS + 0.05% Tween 20), and blocked with 1% BSA in PBS for 30 min at room temperature (RT). Serum or fluid dilutions were assayed as two-fold dilution series in singlicate beginning at 1:1000 for serum and 1:20 for BAL and nasal washes. The biological samples incubated for 2 h at RT were washed with PBS-T and then incubated with of 1:1000 diluted HRP goat anti-mouse IgG (H + L) secondary antibody (Biotium, Fremont, CA, USA) at RT for 1 h. The wells were washed with PBS-T; then, 100 μL of KPL ABTS^®^ Peroxidase Substrate (SeraCare Life Sciences Inc., Milford, MA, USA) was added into each well, and the plates were incubated at RT in the dark for 1 h. Data were collected using the Synergy HT at O.D.405 nm (BioTek Instruments Inc., Winooski, VT, USA). Antibody titers were calculated as the highest serum dilution that registered absorbance values above (≥) the background threshold. The threshold was defined as twice the average value of technical control wells. To ensure reproducibility, intermediate serum timepoints were also tested from tail vein blood extractions and were found to be consistent with the endpoint results (Supplemental Figure S4). A positive control antibody against amino acids 16–30 at 1:1000 dilution was run on every plate to ensure technical consistency (Cat #LS-C829861-100; Lifespan Biosciences, Lynwood, WA, USA).

### 2.7. Lymphocyte Isolation

The mice were euthanized as described above. After fluid collections, the spleens and lungs were collected under sterile conditions. The spleens were harvested and placed in 1X PBS on ice. The lungs were harvested and placed in 1X PBS on ice and then transferred to wells containing 1 mL of digestion buffer (RPMI 1640 media (TFS), 100 μg/mL of Liberase TL (Millipore Sigma), and 100 μg/mL of DNase I (Millipore Sigma)), minced with scissors, and incubated at 37 °C for 30 min. The spleens and lungs were then ground gently with a pestle over 70 μM (spleens) and 40 μM (lungs) mesh filters into 50 mL conical tubes and immediately centrifuged at 300× *g* for 10 min at 4 °C. The supernatant was removed, and the pellet was fully resuspended using 1 mL ACK lysis buffer (Quality Biological, Gaithersburg, MD, USA) and incubated at room temperature (RT) for 3–4 min. To stop cell lysis, the cells were diluted with 30 mL of cold 1X PBS and then pelleted at 300 g for 5 min at 4 °C. The supernatant was again removed, and the remaining pellet was fully resuspended in 5 mL of 1X PBS. After another centrifugation under the same conditions, the supernatant was removed, and the pellet was resuspended in 4 mL (spleens) or 1 mL (lungs) of freezing media (90% FBS (Heat inactivated, Millipore Sigma), 10% DMSO (Fisher Scientific, Hampton, NH, USA), aliquoted into four (spleens) or two (lungs) tubes for cryo-storage using isopropanol cooling containers (Mr. Frosty, TFS, Waltham, MA, USA) at −80 °C for at least 4 h, and then moved to −150 °C. Prior to use, all the cells were counted by a hemocytometer with Gibco™ Trypan Blue solution 0.4% (Life Technologies, Carlsbad, CA, USA).

### 2.8. T-Cell Stimulation and Flow Cytometry

The cryopreserved cells were briefly thawed at 37 °C and slowly diluted to 10 mL with warm complete media (RPMI 1640 (TFS), 10% FBS (Millipore Sigma), 1% penicillin and streptomycin (Gibco™), 20 mM HEPES (TFS), 1% sodium pyruvate (Millipore Sigma), 1% non-essential amino acids (Millipore Sigma), and 1% L-glutamine (TFS)). The cells were spun at 250× *g* for 7 min at room temperature and resuspended to a final concentration of ≤1 × 10^6^ cells per well in 200 µL complete media. The cells were incubated at humid 5% CO_2_ at 37 °C for 2–4 h. The cells were stimulated in duplicate with 1 µg ORF3α (aa. 1-34, GenScript) for 16 h at 37 °C. The positive controls were stimulated for 4 h with 1 µg per well Cell Stimulation Cocktail (Biolegend, San Diego, CA, USA). In the last 4 h of stimulation, 1 µg per well anti-CD28 and anti-CD49d costimulatory antibodies and Brefeldin-A (Biolegend Cat. Nos. 420601, 102116, and 103629) were added to the cells. The cells were transferred to a 96-well V-bottom plate after stimulation, centrifuged 300× *g* for 5 min at room temperature, and washed with 150 µL FACS buffer (0.5% BSA in 1X PBS, Sigma-Aldrich, St. Louis, MO, USA). The pellets were stained with Live/Dead (1:2000 dilution in 1X PBS, LIVE/DEAD Fixable Near-IR Dead Cell Stain Kit, TFS) stain with 100 µL per well and incubated in the dark at room temperature for 30 min. The cells were centrifuged and washed with 150 µL FACS buffer. Then, 50 µL 2% Fc Block (TruStain FcX, Biolegend Cat. No. 101320) was added to each well and incubated on ice in the dark for 15 min. After centrifugation, the cells were resuspended in an anti-mouse extracellular stain cocktail (1:200 FITC-conjugated anti-CD4, 1:200 PercPCy5.5-conjugated anti-CD3, and 1:200 Alexa Fluor 700-conjugated anti-CD8 (Biolegend Cat. Nos. 100405, 100217, and 155022) in FACS) and incubated the dark for 20 min at room temperature. After centrifugation, the cell pellets were resuspended in 150 µL fixation buffer (Cyto-Fast Fix/Perm Buffer Set, Biolegend, Cat. No. 426803) and incubated overnight at 4 °C. The fixed cells were centrifuged at 500× *g* for 5 min at room temperature before staining with 50 µL intracellular stain (1:100 PE-conjugated anti-IL2, 1:200 PECy and conjugated anti-TNF-α, 1:100 APC-conjugated IFN-y (Biolegend Cat. Nos. 506323, 503808, and 505809) in 1X Perm Buffer) for 20 min in the dark at room temperature. Then, 100 µL perm buffer was added after incubation, and the plate was spun down at 500× *g* for 5 min at room temperature. The pellets were washed with 150 µL FACS before resuspension in 150 µL 1X PBS. Flow cytometry was run on an Attune™ NxT Flow Cytometer (TFS). Flow data were analyzed using FlowJo software (FlowJo10.10.0, LLC, Ashland, OR, USA). Gates were formed based on negative stimulation controls and FMO staining controls. The gating strategy is shown in Supplemental Figure S5.

### 2.9. Genotype Analysis and Statistics

Using the ancestral Wuhan virus as the phylogeny root, genotype diversity was analyzed using publicly available Nextstrain (nextstrain.org, URL accessed 2 January 2025) SARS-CoV-2 resources, developed by Sagulenko et al. and Hadfield et al., displaying the observed genetic drift of the SARS-CoV-2 genome (0–29 k bps) from data on all strains collected between December 2019 and January 2025 [34,35].

The datasets comparing three groups were tested by one-way ANOVA with Tukey’s test. The datasets comparing two groups were analyzed by Welch’s *T*-test if standard deviations were significantly different or Student’s *T*-test if not, as noted in the figure legends. The splenic stimulation data were excluded if there were fewer than 100 CD4+ or CD8+ T cells per well. The splenic stimulation samples were run in duplicate wells, and averages of technical replicates are reported. Mouse weights were analyzed by Area Under the Curve (AUC) analysis, with non-overlapping 95% confidence intervals considered significant. All error bars represent the estimation of the standard error of the mean, and a significance level of α ≤ 0.05 was set for all experiments. GraphPad Prism 10 (San Diego, CA, USA) was utilized for all statistical analyses and figure generation. The processed data are supplied in Supplemental Data File S1.

## 3. Results

### 3.1. Antigen Evolution

**Figure 1:**
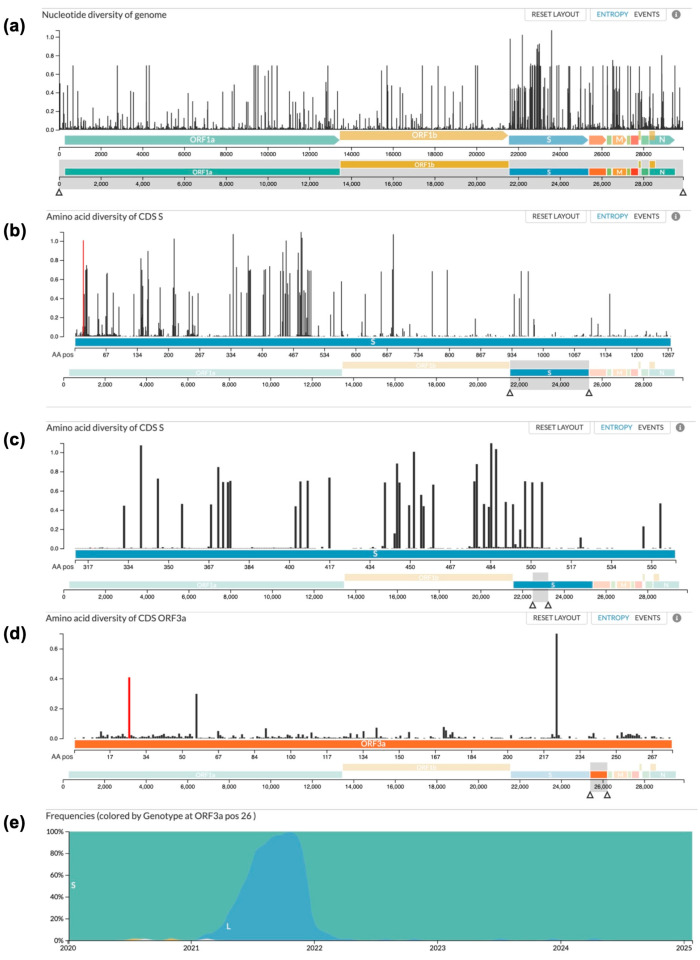
Genotypic diversity comparison across the SARS-CoV-2 genome. (**a**) The complete SARS-CoV-2 genome. Panels (**b**,**c**) show evolutionary changes in amino acid diversity of the (**b**) S region and (**c**) receptor-binding domain (RBD) of the S gene (amino acids 319–541) between December 2019 and January 2025. (**d**,**e**) Evolutionary changes in amino acid diversity of the ORF3a region. Panel (**d**) displays the observed genetic drift across the entirety of the ORF3a region. Panel (**e**) specifically analyzes amino acid 26 for frequencies of amino acid variants over time, with green representing serine (S) and blue leucine (L). The Y-axes display the normalized relative frequency of (**a**) nucleotide or (**b**–**d**) amino acid diversity across the genome, ranging from 0.0 to 1.0.

Utilizing publicly available resources from Nextstrain.org, the genotypic diversity within the SARS-CoV-2 genome was analyzed, showing that most gene regions have diversity over time (Figure 1a). When looking at amino acid sequences, the Spike protein (Figure 1b), the primary target for SARS-CoV-2 vaccines, shows the highest amino acid diversity over time of all the proteins, and the receptor binding domain, the main target of neutralizing antibodies, is the most variable region of all (Figure 1c). In stark contrast, the ectodomain (amino acids 1–36) of the ORF3a protein has proven to be stable since the start of the SARS-CoV-2 pandemic (Figure 1d). Since 2020, only one amino acid within the targeted 6–34 region has changed significantly. As shown in Figure 1e, position 26 of the ORF3a protein remained stable until the beginning of 2021, when B.1.617.2 (Delta) became the primary variant of concern, changing the serine residue to a leucine before reverting to a serine residue in early 2022 after the emergence of the Omicron variant. Importantly, the residue has remained consistent since the reversion. Therefore, this region is an ideal target for vaccines, considering the lack of natural mutations since the origin of the pandemic.

### 3.2. DNA Vaccine Creation

**Figure 2:**
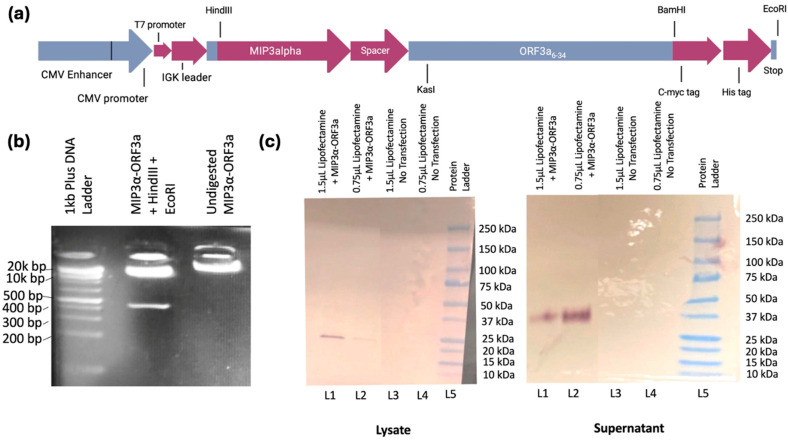
Plasmid design and construct verification. (**a**) Map of the vaccine construct within the pSecTag2b mammalian expression plasmid designed with BioRender.com and Snap Gene (version 8.0) softwares, with full-length human *MIP3α* fused to the *ORF3a_6-34_* region. (**b**) Double digest and undigested form of the vaccine plasmid as further verification of construct purity and correctness. Relevant band sizes are labeled. (**c**) Plasmids were transfected into HEK293T cells with noted volumes of Lipofectamine 3000. Samples of cell lysate and cell supernatant were separated by 4–20% SDS-PAGE, transferred to a nitrocellulose membrane, and blotted for the c-myc peptide tag. Samples were run in duplicate and are representative of three independent transfection trials. Unnecessary lanes were removed from the panel.

The primary hypothesis of this study was to assess whether an immunogenic vaccine targeting the evolutionarily stable ectodomain of ORF3a (amino acids 6–34) could be designed. First, a DNA vaccine was constructed fusing the *ORF3a* genetic domain to the *MIP3α* gene (Figure 2a), which has been studied extensively in our laboratory and has been shown across models to enhance immune responses [17,18,19,20,22,23,24,25].

DNA quality and correctness of the synthesized *MIP3α-ORF3a* DNA fusion vaccine in the mammalian expression vector pSecTag2b was assessed and verified by gel electrophoresis and restriction enzyme analysis (Figure 2b), as well as insert sequencing (Supplemental Data File S2). Lane 2 shows the digest with HindIII and EcoRI results in the expected linear plasmid band and a single band representing the expressed vaccine sequence at the expected size (426 bp), and lane three shows the plasmid vaccine was primarily in a supercoiled state. To ensure that the expression vector was functional in a mammalian cell, the vaccine was transiently transfected into HEK293T cells, and cell lysate and supernatants were collected. Qualitative Western blot analysis of the fusion vaccine targeting the C-terminal myc tag confirmed full-length protein production that was present in the lysate at the expected size (approximately 20 kDa) and secreted into the supernatant likely as a dimer (approximately 40 kDa) (Figure 2c). Previous MIP3α-antigen vaccines from the laboratory have not shown dimerization [21,22,24]. It is probable, therefore, that the ectodomain plays a role in the known oligomerization of ORF3a [4].

### 3.3. DNA Intramuscular Vaccine Immunogenicity

**Figure 3:**
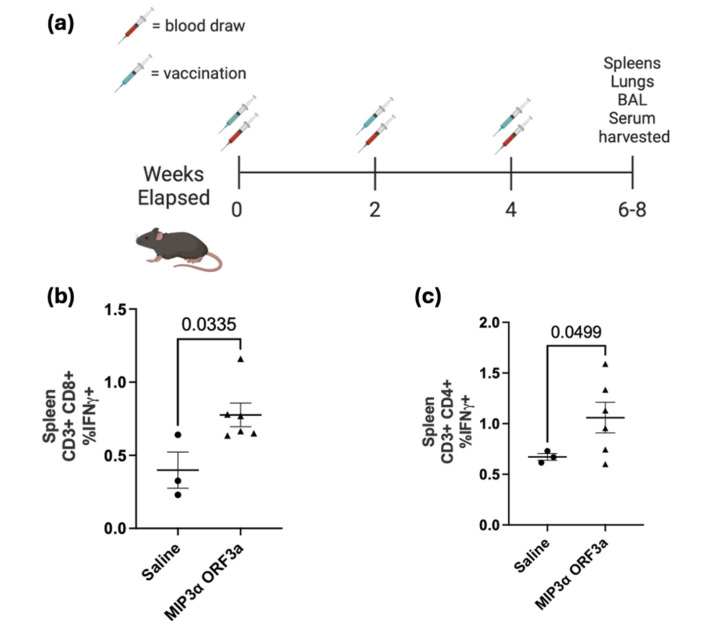
Vaccine immunogenicity therapy schedule and intramuscular vaccination. (**a**) Vaccine immunogenicity study design for all experiments. For the experiment shown in (**b**,**c**), C57BL/6 female mice (n = 3 for the saline group, n = 6 for *MIP3α-ORF3a*) were immunized three times at two-week intervals with 20 µg *MIP3α-ORF3a* DNA. Three weeks after the 3rd immunization, the vaccinated mice were euthanized, and their spleens were harvested. The percentage of (**b**) CD8+ or (**c**) CD4+ T cells that were positive for IFN-γ in the spleen were analyzed after being stimulated with 1 μg of SARS-CoV-2 ORF3a_1-34_ peptide. A Student’s *T*-test was performed on the CD8+ population dataset, and a Welch’s *T*-test was performed on the CD4+ population dataset to account for the dissimilar standard deviations between the two groups.

Utilizing a standard intramuscular electroporation vaccination schedule in a mouse model that has shown efficacy in prior work [24] (Figure 3a), the immunogenicity of the DNA vaccine was tested [32,33]. Three weeks after the third vaccination, tissues were collected. The ELISA assays of serum showed insignificant antibody responses (Supplemental Figure S6). The splenocytes were collected and then stimulated with ORF3a peptide in the presence of cell transport inhibitors, followed by staining with surface antibodies and intracellular staining for T-cell activation cytokines for analysis by flow cytometry. The lymphocytes were gated for populations of CD4- and CD8-positive T cells and then assessed for IFN-γ and TNF-α production. Our results demonstrated a significant increase (*p* < 0.05) in CD8+ T cell splenocytes that were positive for IFN-γ between the saline group and the *MIP3α-ORF3a*-vaccinated group, showing an almost two-fold difference (0.4% vs. 0.78%) (Figure 3b). The *MIP3α-ORF3a*-vaccinated group also demonstrated a significant increase (*p* < 0.05) in splenocytes that were positive for IFN-γ-producing CD4+ T cells, showing an increase of 58% over the background level (Figure 3c). The splenocytes did not show increases in TNF-α under the conditions tested. Pilot experiments with adjuvants, also given intramuscularly, showed that CpG types B and C did not dramatically increase the T-cell response over the known immunostimulatory activity of electroporation [33,36,37], but the addition of a STING agonist did increase T-cell responses (Supplemental Figure S7). However, the mice receiving the STING agonist lost weight over time, so the experiment was not repeated due to suspected toxicity (Supplemental Figure S2d).

### 3.4. DNA Intranasal Vaccine Immunogenicity

**Figure 4:**
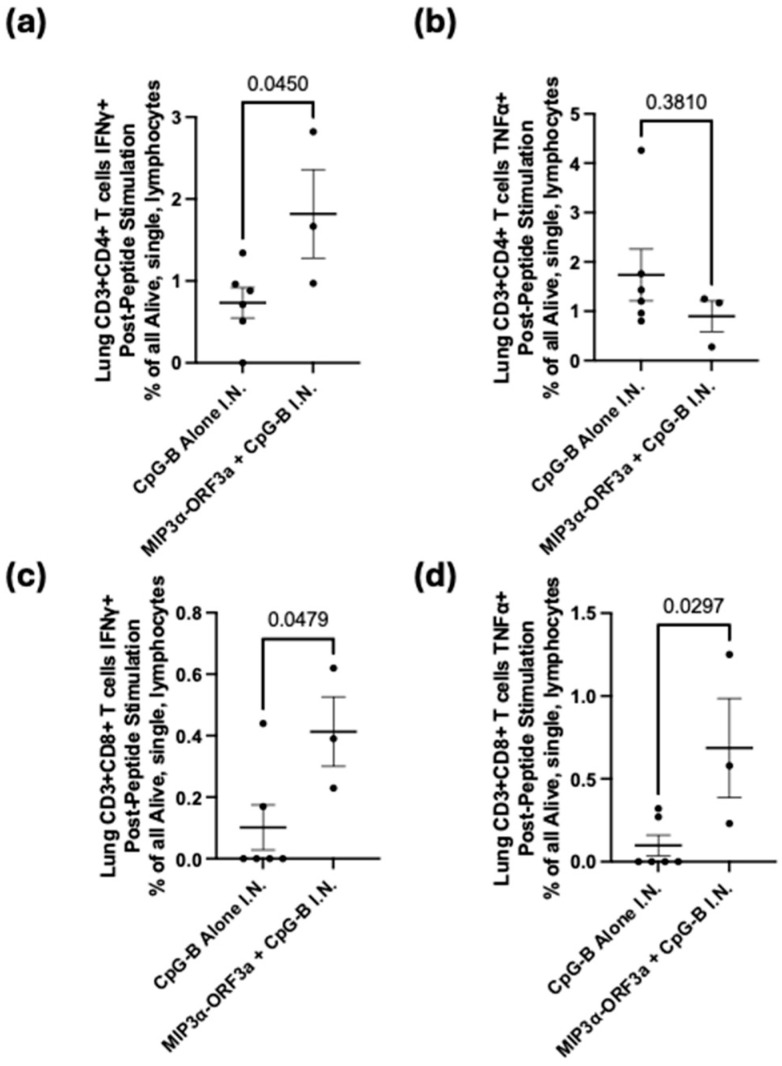
Intranasal DNA vaccination. C57BL/6 mice (n = 6 [3M, 3F] for the CpG-B alone group; n = 3 [1M, 2F] for *MIP3α-ORF3a* + CpG-B) were immunized intranasally three times at two-week intervals with 20 µg of CpG-B with or without 200 µg *MIP3α-ORF3a* in the same volume. Two weeks after the 3rd immunization, the mice were euthanized, their lungs were harvested, and the cells were stimulated with the vaccine peptide. Percentages of total live, single lymphocytes are shown for CD4 (**a**,**b**)- and CD8 (**c**,**d**)-positive T cells expressing IFN-γ (**a**,**c**) and TNF-α (**b**,**d**). Significance was determined by Student’s *T*-Test.

A previous DNA vaccine fusing *MIP3α* to the SARS-CoV-2 Spike receptor binding domain sequence proved that intranasal forms of our DNA vaccine could induce lung-associated T-cell responses [24]. It was hypothesized that the *MIP3α-ORF3a* vaccine would similarly be able to induce a T-cell response at the lung site of potential infection. To assess T-cell specificity for ORF3a after intranasal vaccination with the *MIP3α-ORF3a* DNA vaccine construct, the mice were immunized in the same schedule (Figure 3a) but intranasally. To enhance responses for an intranasal DNA vaccine unable to receive electroporation, CpG-B adjuvant was mixed with the vaccine DNA. Tissues were collected two weeks after the final vaccination. The lungs were processed into single cells and stimulated as before. Previous work has shown that MIP3α vaccines administered intranasally were especially potent at recruiting more overall effector T cells to the lung, so the most informative measure would be to assess responses as an overall percentage of cells and not a percentage of T cells [24]. The CD4+ and CD8+ T cell populations both demonstrate significant (*p* < 0.05) differences between the adjuvant-only and adjuvant with DNA sample groups, with increased IFN-γ production in the samples given the vaccine as well as the adjuvant (Figure 4a,c). There is also a significant (*p* < 0.05) increase in TNF-α production by the CD8+ T cells, but this is not seen in the CD4+ T cells (Figure 4b,d). As demonstrated by cytokine production from the CD4+ and CD8+ T cells, the intranasally administered vaccine elicits a significant T-cell response in the critical site of the lung.

### 3.5. ORF3a-KLH Peptide Vaccine

**Figure 5:**
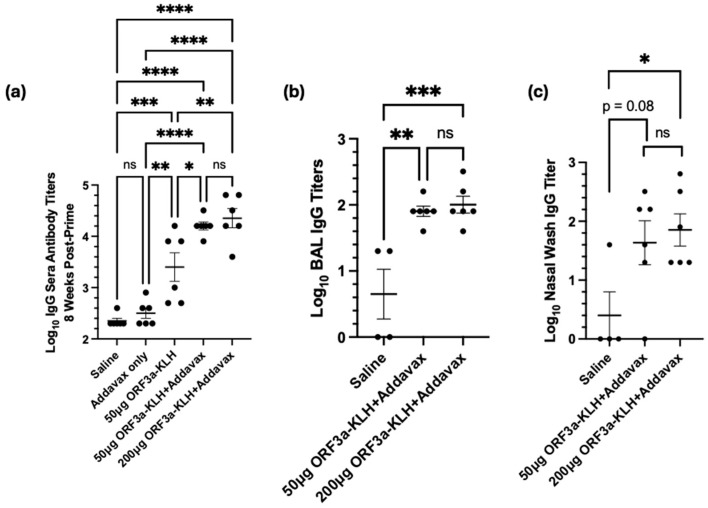
Peptide vaccination. The BALB/C mice (n = 6; 3M, 3F) were immunized intraperitoneally three times at two-week intervals with the amounts noted in the figure axes for ORF3a-KLH, with or without an equal volume mixture of Addavax adjuvant. Four weeks after the 3rd immunization, samples were collected. Antibody titers were recorded for (**a**) serum, (**b**) bronchoalveolar lavage (BAL), and (**c**) nasal wash fluids. Significance was determined by one-way ANOVA with Tukey’s test for multiple comparisons. Due to poor fluid yields, one male and one female mouse each from the control group could not be analyzed for BAL and nasal wash assays. * *p* < 0.05, ** *p* < 0.01, *** *p* < 0.001, and **** *p* < 0.0001.

We hypothesized that the specific formulation of the DNA vaccine was responsible for the lack of antibody response and not a biological trait of the region. The specific sub-region of the ectodomain ORF3a_15-28_, known to elicit an antibody response in SARS-CoV-1 systems [12,38,39], was created in peptide form fused to immunogenic carrier protein Keyhole limpet hemocyanin (KLH). With the same schedule as Figure 3a, the mice were immunized intraperitoneally with ORF3a-KLH peptide along with Addavax adjuvant. To quantify antibody production in response to vaccine administration, sera, BAL fluid, and nasal wash fluid were collected four weeks after the third vaccination and were assessed by ORF3a-specific antibody titers. Titers from the sera (Figure 5a) showed that without adjuvant, the vaccine resulted in titers roughly one log above the background (*p* < 0.01). The addition of Addavax adjuvant further boosted the titer by almost an additional order of magnitude (*p* < 0.05, compared to without adjuvant). Interestingly, increasing the dose to 200 μg of ORF3a-KLH with Addavax did not result in higher titers as compared to 50 μg.

Mucosal fluids were also tested to assess the presence of antibodies at the primary site at which SARS-CoV-2 infection and disease would be manifest. The BAL titers show significantly higher antibody levels from animals receiving the ORF3a-KLH with Addavax vaccine than in the negative control animals receiving saline. Similar to the serum, no difference is seen between the doses of 50 µg or 200 µg ORF3a-KLH (Figure 5b). The nasal wash samples also provide data showing vaccine-induced antibody responses, with a significant difference (<0.05) between the saline and the 200 µg ORF3a-KLH and Addavax groups, with a trend toward a significant difference (*p* = 0.08) between the saline and the 50 µg ORF3a-KLH and Addavax groups (Figure 5c). The consistent trend seen across all the collected sample sources indicates that ORF3a-KLH administered with Addavax effectively elicits an antibody response systemically and in the mucosa.

## 4. Discussion

The viability of the ORF3a’s ectodomain (amino acids 1–36) as a well-conserved target for SARS-CoV-2 was assessed in terms of genetic drift across variants over the past several years, as visualized in Figure 1d. The amino acid composition is surprisingly well conserved across the SARS-CoV-2 variants, with only amino acid 26 being variable, starting as serine before changing to leucine with the Delta strain and then back to serine with the Omicron strain (Figure 1e). With this high level of conservation in the ORF3a ectodomain demonstrated across variants, as expected based on the previous literature, we believe that ORF3a presents a vaccine target that would be less susceptible to mutational escape [5,40,41]. Given the challenges of targeting the highly variable Spike protein, mutations that have decreased the long-term efficacy of the available vaccines, ORF3a may be a more effective target that could increase the window of efficacy for COVID-19 vaccines [5,40,41,42,43].

Fusing *MIP3α* and *ORF3a* in a DNA vaccine elicits a significant cell-mediated immune response. With both intramuscular (IM) administration (with electroporation) and intranasal (IN) administration (with CpG adjuvant), *MIP3α-ORF3a*-vaccinated mice elicited significant specific CD4+ and CD8+ T-cell responses, as measured by ex vivo cytokine production post-stimulation of T cells from the spleen (IM) and lung (IN) (Figure 3 and Figure 4) [44]. This is the first study, to our knowledge, showing the feasibility of eliciting a T-cell response to SARS-CoV-2 ORF3a. However, the DNA vaccine was unable to induce antibody responses to ORF3a_6-34_. One potential explanation is that the apparent dimerization of the induced ORF3a_6-34_ protein from the DNA vaccine (Figure 2c) could result in steric hindrances or other protein interactions that result in inefficient binding to the B-cell receptor complex and antibodies, but these would not be an impediment to the induction of T-cell responses post-antigen processing. Importantly, though, the intranasal version of the DNA vaccine was able to elicit robust T-cell responses within the lung environment, which are known to be important for limiting disease severity [6,7]. Furthermore, T-cell immunity is less subject to genetic drift escape if a rare mutation event in this domain does happen [9,10,11].

Therefore, a second formulation was designed to investigate whether an antibody response to this region could be induced by a vaccine. Instead of DNA, a peptide vaccine comprising a stable region of the ectodomain (ORF3a_15-28_) was constructed and fused to an immunogenic carrier molecule, Keyhole limpet hemocyanin (KLH) [38,45]. Immunogenicity mouse studies with ORF3a-KLH showed robust antibody responses induced with the combination of the vaccine with Addavax adjuvant (Figure 5). Antibodies were detected in the serum, lung lavage fluid, and nasal wash samples, suggesting that the immune response included the mucosal sites of initial infection. Interestingly, this vaccine was unable to elicit robust T-cell responses in the lung (Supplemental Figure S8).

Safety is paramount in prophylactic vaccine studies. We utilized mouse weight as a measure of systemic toxicity. One adjuvant protocol showed signs of the mice losing weight and was, therefore, discontinued, but all other protocols presented resulted in steady mouse weight gain (Supplemental Figure S2). However, ORF3a has been shown to sensitize cells to ferroptosis, which plays a role in tissue and organ damage during infection [46]. Since we utilized only the ectodomain and not full-length protein, we hypothesize that our vaccine will not cause adverse reactions, but this will be a top priority for future work.

## 5. Conclusions

The success of these vaccine models in developing adaptive immune responses to the highly conserved SARS-CoV-2 ORF3a ectodomain supports our hypothesis that ORF3a could be a viable vaccine target. While the lack of a challenge model is a limitation of this study, we hypothesize that combining the intranasal administration of the DNA vaccine with the systemic administration of the peptide vaccine would elicit both specific T-cell and antibody responses in the mucosal lung environment that could provide protection and/or disease mitigation for the host. Further work should also be performed to utilize longer intervals between vaccinations, test vaccines without MIP-3α to confirm enhancement, compare other formulations, such as liposome-coated mRNA, which might induce more balanced responses, and test this combination in an animal challenge model system [47,48].

## Data Availability

The presented data are provided in the submission supplement. Any other data or materials are available upon reasonable request.

## Supplementary Materials

The following supporting information can be downloaded at https://www.mdpi.com/article/10.3390/vaccines13030220/s1: Figure S1: Unedited Blots; Figure S2: Mouse weights over time post vaccination; Figure S3: *MIP-3α-ORF3a* DNA vaccine with (MCovPADRE) and without (MCov) PADRE sequence; Figure S4: ORF3a-KLH antibody titers over time; Figure S5: Flow gating strategy; Figure S6: ELISA dilutions testing sera from *Mip3α-ORF3a* immunized mice with (MipCovPadre) or without (MipCov) the PADRE tag at labeled time points; Figure S7: Intramuscular immunization with adjuvant pilot; Figure S8: Intracellular Cytokine Staining analysis of ex vivo orf3a ectodomain-stimulated lymphocytes isolated from harvested lung tissue of animals vaccinated with the ORF3a-KLH peptide vaccination series; Data S1: Dataset; Data S2: DNA vaccine sequence.

## Abbreviations

The following abbreviations are used in this manuscript:

SCV2	SARS-CoV-2
ORF	open reading frame
MIP3α	macrophage-inflammatory protein 3 alpha
IM	intramuscular
IN	intranasal
KLH	keyhole limpet hemocyanin
iDC	immature dendritic cell
IFNγ	interferon gamma
TNFα	tumor necrosis factor alpha
STING	stimulator of interferon genes
BAL	bronchoalveolar lavage
