# Supplementary material for: Novel Vaccines Targeting the Highly Conserved SARS-CoV-2 ORF3a Ectodomain Elicit Immunogenicity in Mouse Models
Source: Vaccines (Basel). 2025 Feb 22;13(3):220. doi: 10.3390/vaccines13030220 (PMC11946519; doi:10.3390/vaccines13030220)
Supplement: Supplementary file 1 [file vaccines-13-00220-s001.zip › Supplemental Figure legends.pdf]

Supplemental Figure S1: Unedited blots. As noted in the figure, lanes not relevant to this paper were removed for Figure 2, and images were spliced only with pieces of the same blot, keeping the same relative orientations.

Supplemental Figure S2. Mouse weights over time post vaccination. **(a)** Mouse weight change over time after vaccination with *MIP3α-ORF3a* DNA vaccine (MipCov) or saline control. **(b)** Mouse weight change after intranasal vaccination with CpG Type B and 200µg *MIP3α-ORF3a* DNA vaccine. **(c)** Mouse weight change after vaccination with ORF3a-KLH protein vaccine and Addavax. **(d)** Mouse weight change after intramuscular vaccination with *MIP3α-ORF3a* DNA vaccine and varying adjuvants. Significance determined by Area Under the Curve (AUC) analysis.

Supplemental Figure S3. *MIP-3α-ORF3a* DNA vaccine with (MCovPADRE) and without (MCov) PADRE sequence**. (a)** CD8 and **(b)** CD4 IFN-γ production analysis comparing the PADRE formulation to the *MIP-3αORF3a* vaccine. Differences were analyzed by Student's T-test.

Supplemental Figure S4: ORF3a-KLH antibody titers over time. Tested by area under the curve, with non-overlapping 95% confidence intervals considered significant. †significant compared to all three control groups: 50ug vaccine only, Addavax only, and saline. #significant only compared to saline.

Supplemental Figure S5. Flow gating strategy

Supplemental Figure S6: ELISA dilutions testing sera from *Mip3α-ORF3a* immunized mice with (MipCovPadre) or without (MipCov) the PADRE tag at labeled time points.

Supplemental Figure S7. Intramuscular immunization with adjuvant pilot. T cells isolated from the spleens of mice vaccinated with *MIP3α-ORF3a* DNA vaccine with electroporation alongside an adjuvant, either CpG Type B, CpG Type C, or STING agonist. CD4+ T cells expressing **(a)** IFN-γ, **(b)** TNF-α or **(c)** IL-2 and CD8+ T cells expressing **(d)** IL-2, **(e)** TNF-α or **(f)** IFN-γ were analyzed. Significance was determined by ANOVA with Tukey's multiple comparisons test.

Supplemental Figure S8: Intracellular Cytokine Staining analysis of ex vivo orf3a ectodomain-stimulated lymphocytes isolated from harvested lung tissue of animals vaccinated with the ORF3a-KLH peptide vaccination series.
