# Supplementary figures and images for: Novel Vaccines Targeting the Highly Conserved SARS-CoV-2 ORF3a Ectodomain Elicit Immunogenicity in Mouse Models
Source: Vaccines (Basel). 2025 Feb 22;13(3):220. doi: 10.3390/vaccines13030220 (PMC11946519; doi:10.3390/vaccines13030220)

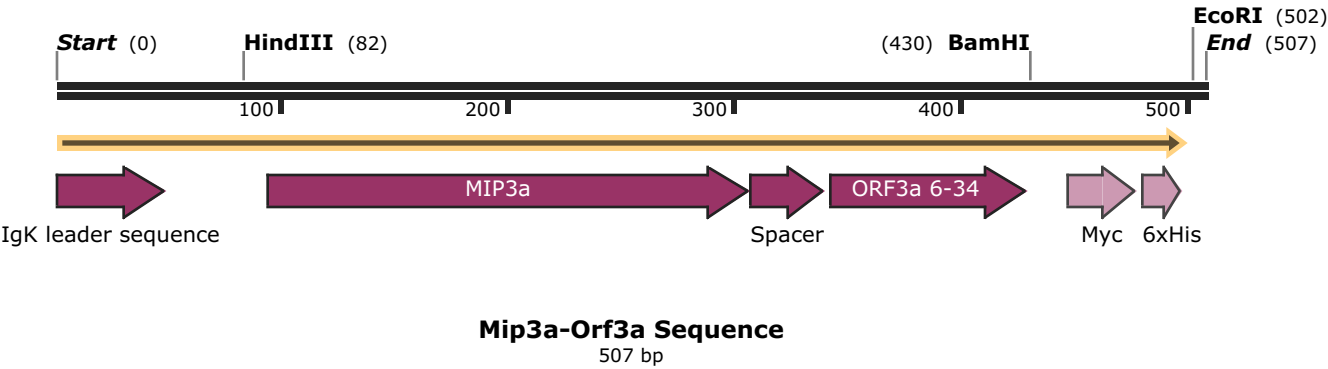

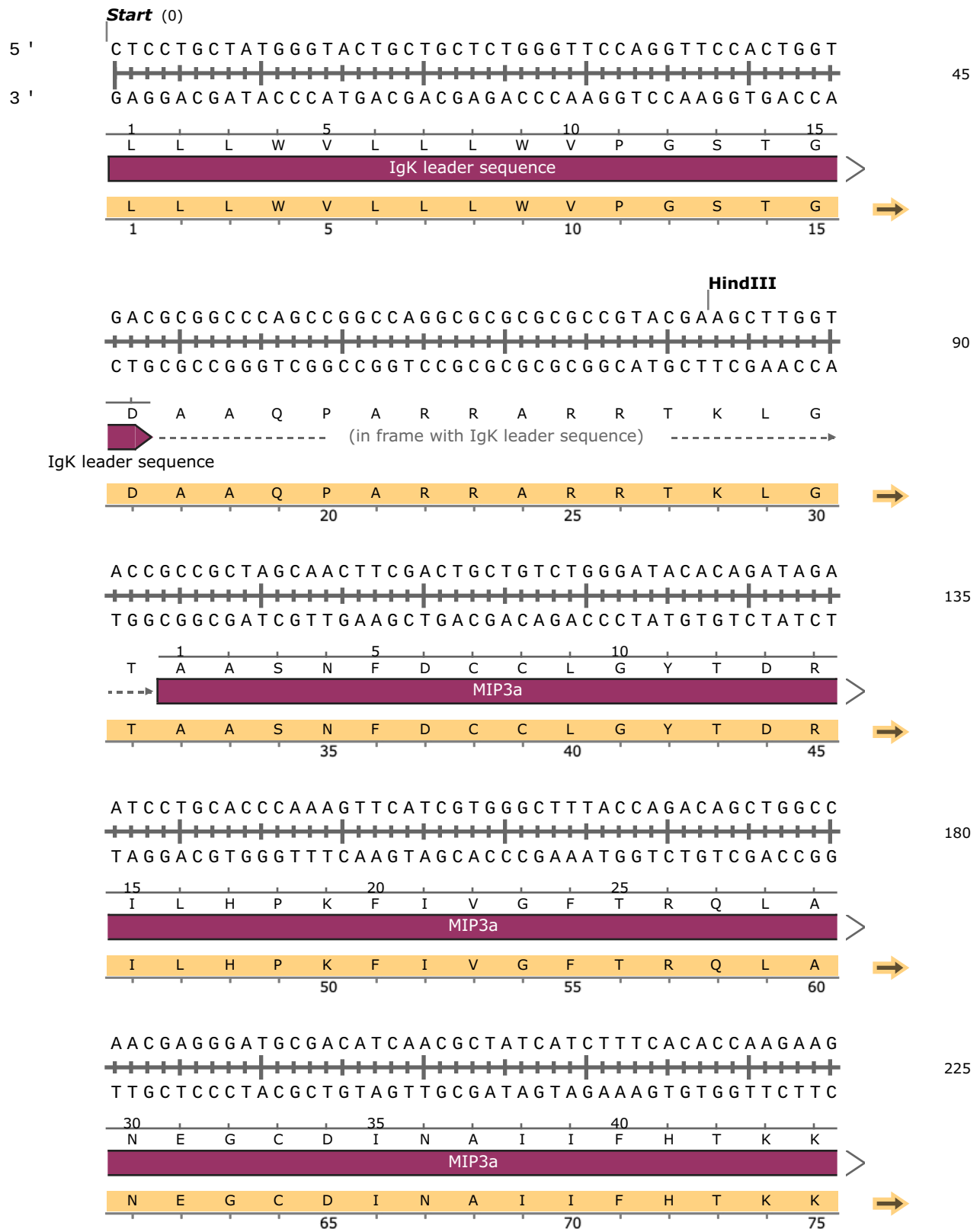

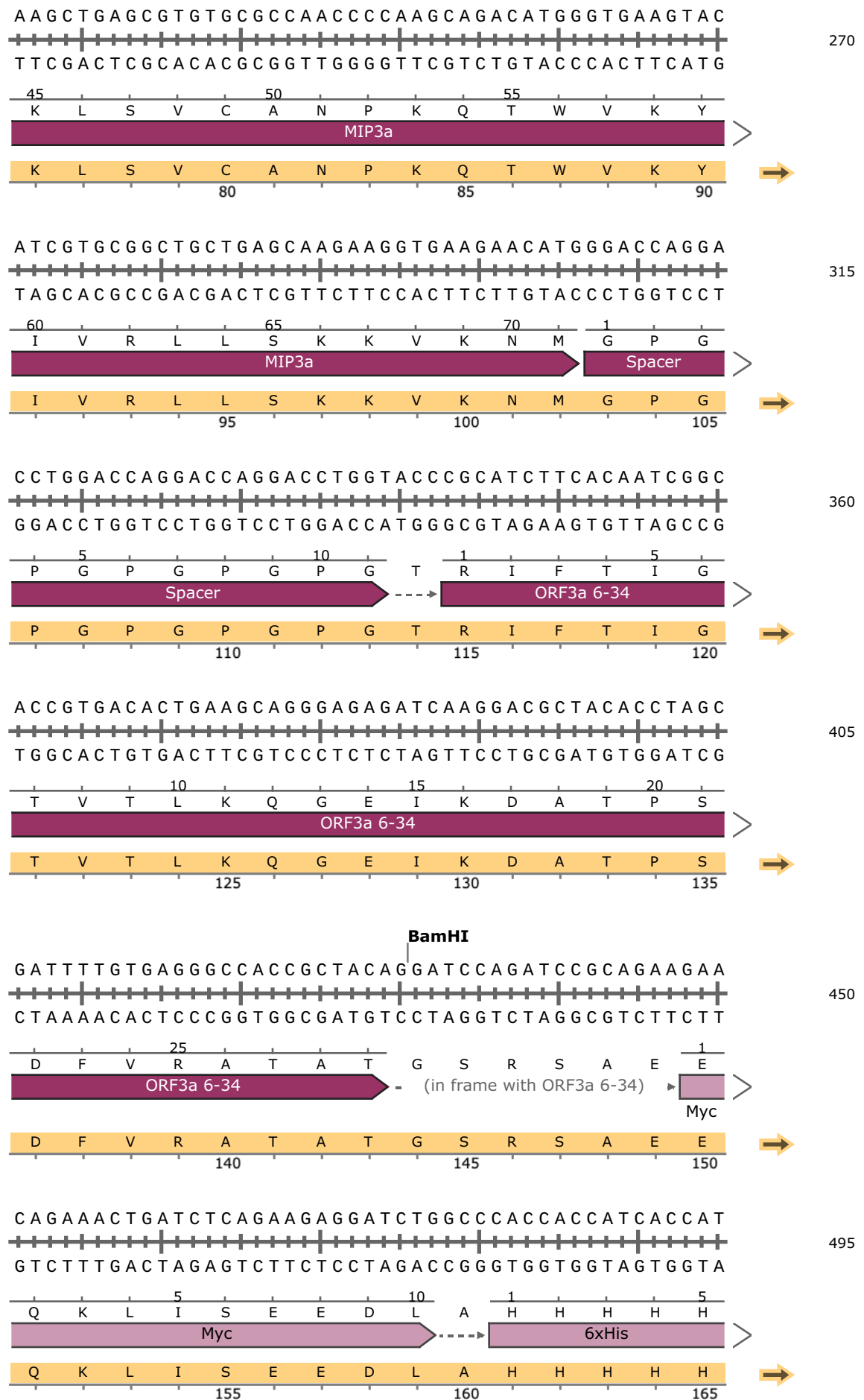

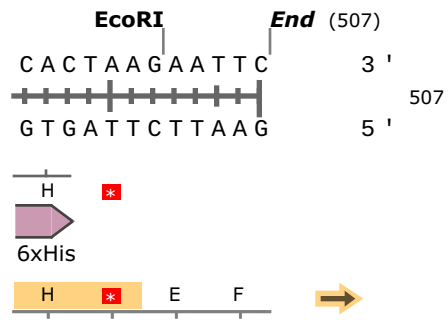

Supplement: Supplementary file 1 [file vaccines-13-00220-s001.zip › Data S2. Mip3a-Orf3a Sequence.pdf]

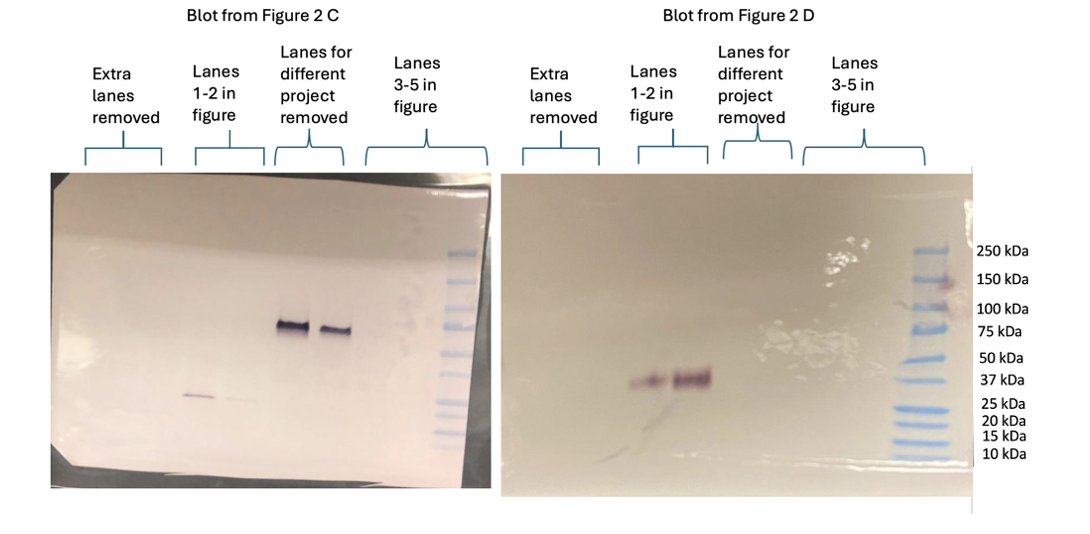

Supplement: Supplementary file 1 [file vaccines-13-00220-s001.zip › Figure S1.tiff]

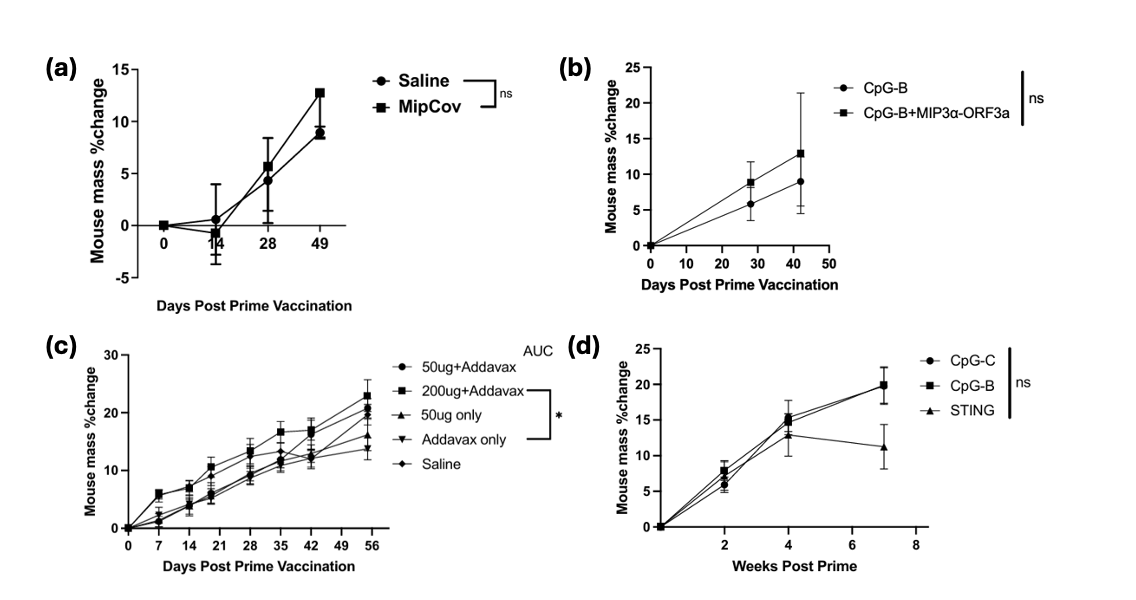

Supplement: Supplementary file 1 [file vaccines-13-00220-s001.zip › Figure S2.tiff]

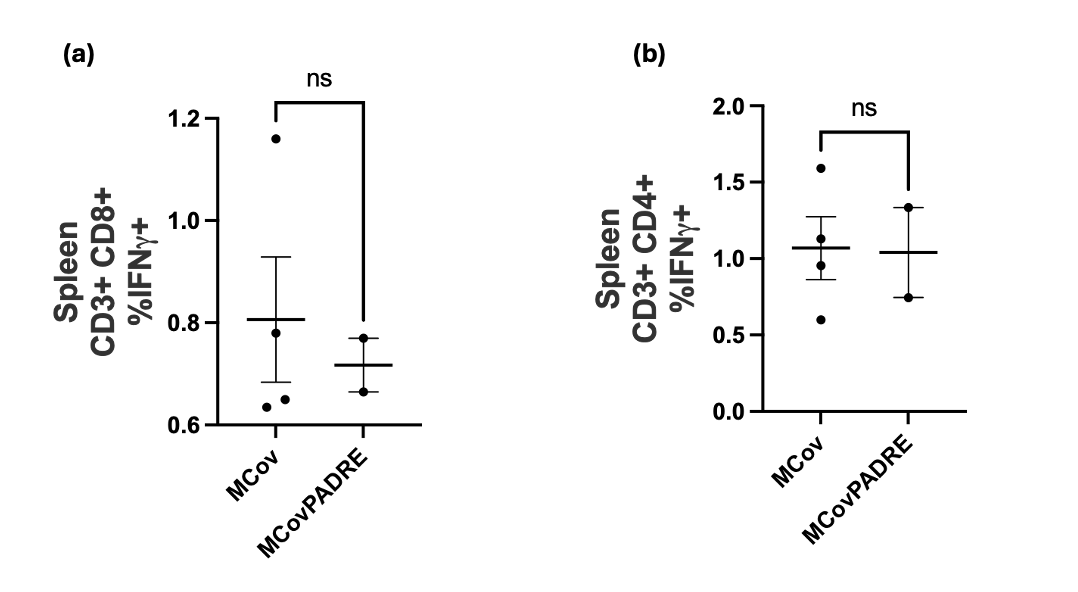

Supplement: Supplementary file 1 [file vaccines-13-00220-s001.zip › Figure S3.tiff]

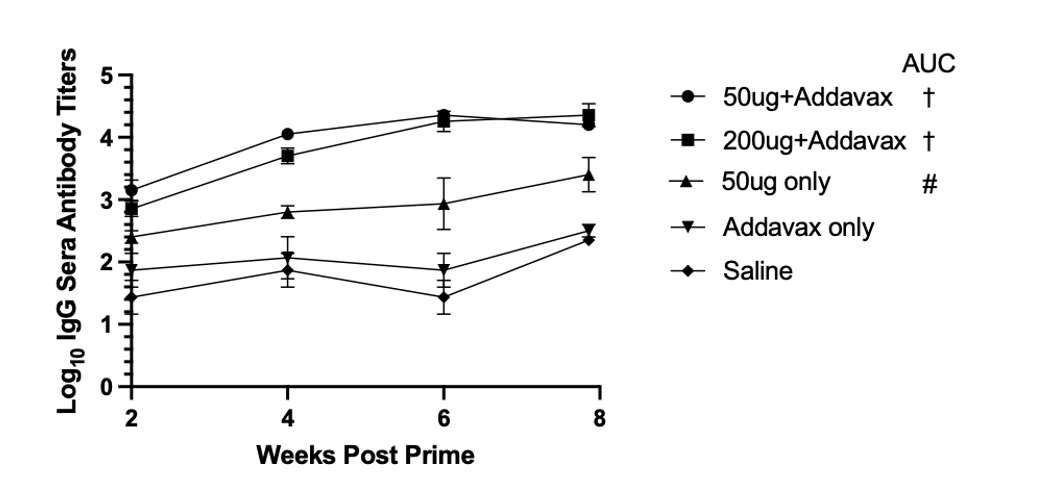

Supplement: Supplementary file 1 [file vaccines-13-00220-s001.zip › Figure S4.tiff]

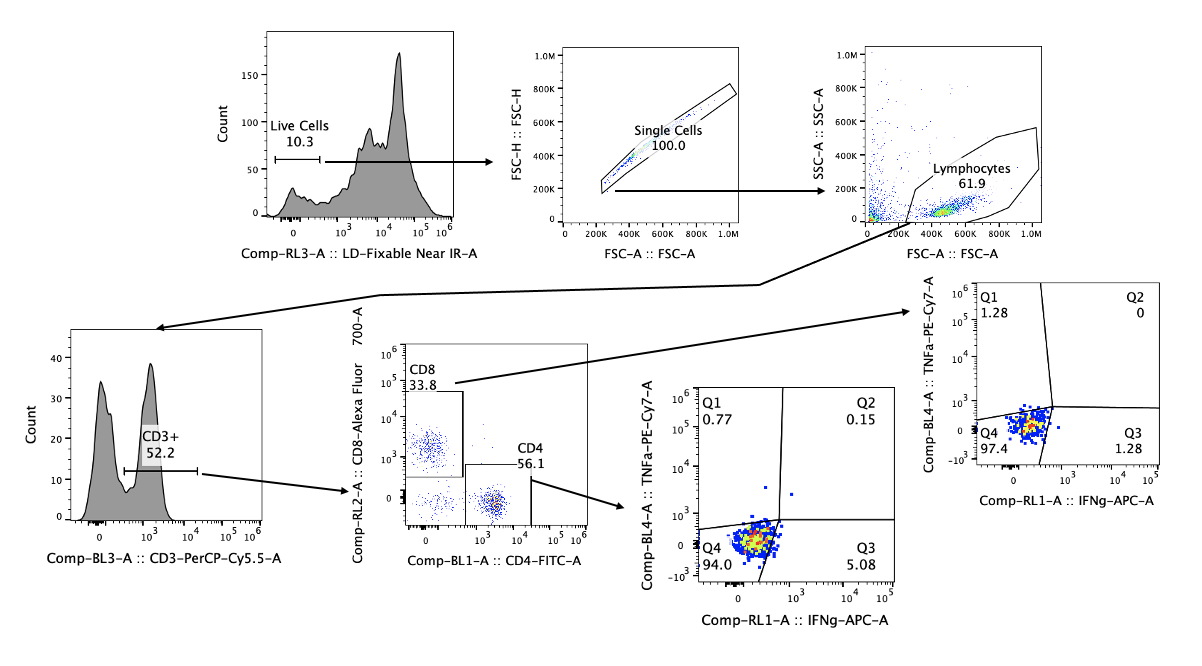

Supplement: Supplementary file 1 [file vaccines-13-00220-s001.zip › Figure S5.tiff]

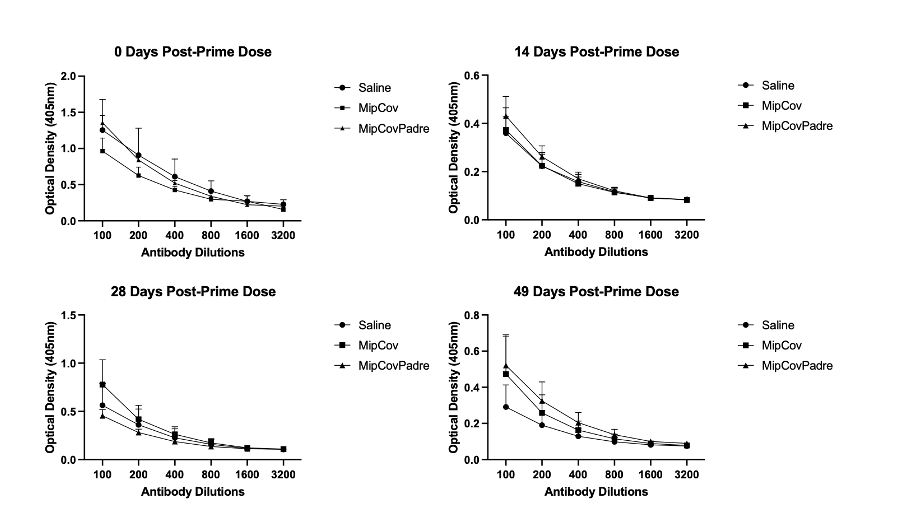

Supplement: Supplementary file 1 [file vaccines-13-00220-s001.zip › Figure S6.tiff]

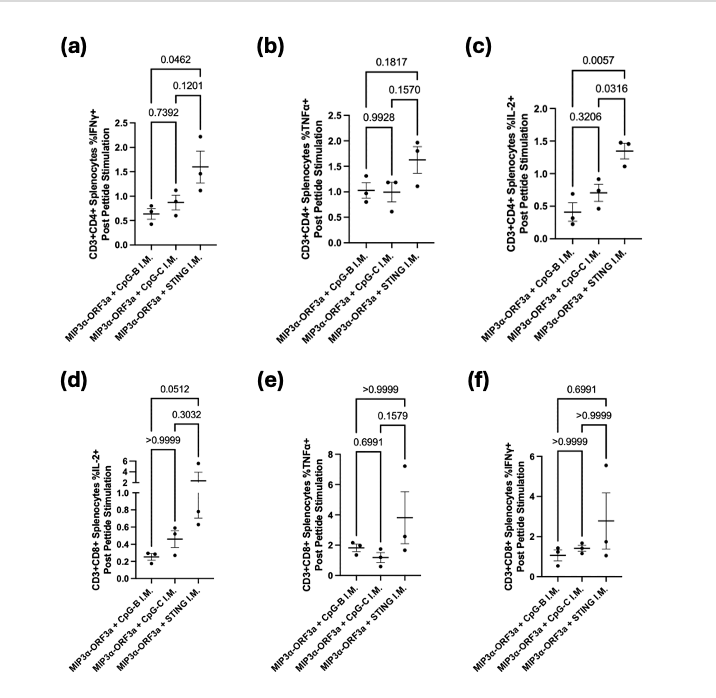

Supplement: Supplementary file 1 [file vaccines-13-00220-s001.zip › Figure S7.tiff]

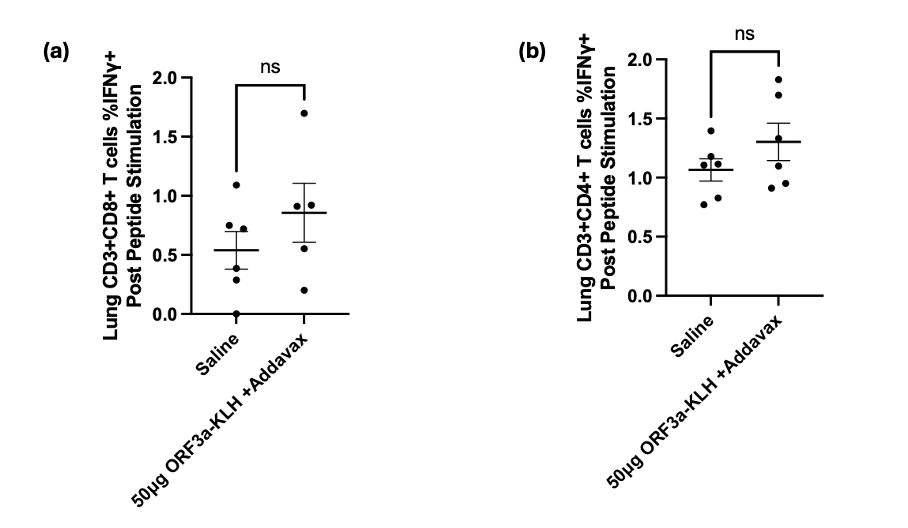

Supplement: Supplementary file 1 [file vaccines-13-00220-s001.zip › Figure S8.tiff]
